# Modulation of Insulin Amyloid Fibrillization in Imidazolium-Based Ionic Liquids with Hofmeister Series Anions

**DOI:** 10.3390/ijms24119699

**Published:** 2023-06-02

**Authors:** Vladimir Vanik, Zuzana Bednarikova, Gabriela Fabriciova, Steven S.-S. Wang, Zuzana Gazova, Diana Fedunova

**Affiliations:** 1 Institute of Experimental Physics, Slovak Academy of Sciences, 040 01 Košice, Slovakia; vanik@saske.sk (V.V.); bednarikova@saske.sk (Z.B.); 2 Department of Biophysics, Faculty of Science, Pavol Jozef Šafárik University in Košice, 041 54 Košice, Slovakia; gabriela.fabriciova@upjs.sk; 3 Department of Chemical Engineering, National Taiwan University, Taipei 10617, Taiwan; sswang@ntu.edu.tw

**Keywords:** ionic liquids, amyloid aggregation, Hofmeister series, amyloid fibril morphology

## Abstract

Amyloid fibrils have immense potential to become the basis of modern biomaterials. The formation of amyloid fibrils in vitro strongly depends on the solvent properties. Ionic liquids (ILs), alternative solvents with tunable properties, have been shown to modulate amyloid fibrillization. In this work, we studied the impact of five ILs with 1-ethyl-3-methylimidazolium cation [EMIM^+^] and anions of Hofmeisterseries hydrogen sulfate [HSO_4_^−^], acetate [AC^−^], chloride [Cl^−^], nitrate [NO_3_^−^], and tetrafluoroborate [BF_4_^−^] on the kinetics of insulin fibrillization and morphology, and the structure of insulin fibrils when applying fluorescence spectroscopy, AFM and ATR-FTIR spectroscopy. We found that the studied ILs were able to speed up the fibrillization process in an anion- and IL-concentration-dependent manner. At an IL concentration of 100 mM, the efficiency of the anions at promoting insulin amyloid fibrillization followed the reverse Hofmeister series, indicating the direct binding of ions with the protein surface. At a concentration of 25 mM, fibrils with different morphologies were formed, yet with similar secondary structure content. Moreover, no correlation with the Hofmeister ranking was detected for kinetics parameters. IL with the kosmotropic strongly hydrated [HSO_4_^−^] anion induced the formation of large amyloid fibril clusters, while the other kosmotropic anion [AC^−^] along with [Cl^−^] led to the formation of fibrils with similar needle-like morphologies to those formed in the IL-free solvent. The presence of the ILs with the chaotropic anions [NO_3_^−^] and [BF_4_^−^] resulted in longer laterally associated fibrils. The effect of the selected ILs was driven by a sensitive balance and interplay between specific protein–ion and ion–water interactions and non-specific long-range electrostatic shielding.

## 1. Introduction

Amyloid fibrils have attracted great interest from outside the medical field, expanding their potential use to the material sciences. Their unique physicochemical properties, such as high stability, strength, elasticity, and resistance against degradation, allow the creation of new progressive biomaterials based on amyloid fibrils and open various applications in nanotechnology and artificial functional materials [1,2,3,4]. Moreover, adhesion to solid interfaces of amyloid fibrils can be efficiently exploited to produce layered nanocomposites, with graphene, carbon, and metal nanocomposites with various amyloid fibrils having already been described [5] as well as amyloid-based biosensors [6] and amyloid-templated optoelectronic materials [7]. One of the most rapidly emerging applications for amyloid fibrils is their use as scaffolds to promote and control cell growth, proliferation, and differentiation of epithelial cells and fibroblast as 2D fibrillar networks, films, or fibrils decorated by biocompatible polymers [8].

Therefore, there are a vast number of studies focusing on finding strategies to specifically regulate the protein self-assembly process in vitro to optimize amyloid fibril production. The knowledge obtained may also contribute to and expand the formulation and definition of the fundamental principles of amyloid self-assembly.

It is generally recognized that the protein’s ability to self-assemble into amyloid fibrils is the intrinsic property of the polypeptide chain [9,10]. Despite their different chemical natures and origins, amyloid fibrils formed from different proteins have a set of common characteristics [11,12,13,14].

The resulting structure of amyloid fibrils is closely related to the conditions under which the fibrillization occurred. Moreover, depending on conditions, the same protein sequence can produce morphologically different fibrils [13,15]. The specific conditions required for fibril formation in vitro are usually achieved by adjusting temperature, pH, ionic strength, adding denaturants or altering the solvent composition.

Insulin is a 51-residue hormone involved in regulating the blood glucose level and is used to treat diabetes. It is considered a model protein with a well-known sequence and structure. It has been shown that insulin exhibits an in vitro amyloid fibril-forming tendency under certain destabilizing conditions, with the resulting amyloid fibrils having a variety of morphologies, depending on the protein concentration, pH, temperature, agitation, solvent composition, ionic strength, and the presence of stabilizers or denaturants [13,16,17]. Moreover, insulin fibrils can be prepared in a manageable time and in low concentrations at acidic pH. This variability can be further explored for utilizing amyloid fibrils as biomaterials.

Ionic liquids (ILs) represent a new class of solvents consisting entirely of ions (large organic cation combined with inorganic/organic anion). By selecting the type of ions, the composition of ILs can be designed to match the desired physicochemical properties (melting temperature, polarity, hydrophobicity, density, viscosity, solubility, and more) for the selected applications [18,19]. ILs can be used neat or diluted in water or other solvents. Several studies have reported that ILs can also effectively promote, but also inhibit, the formation of amyloid fibrils from various proteins or even dissolute amyloid aggregates [20,21,22,23,24,25,26]. Kumar and Venkatesu found that ammonium-based protic ILs stabilize native insulin at elevated temperatures and prevent insulin self-aggregation [27]. Several research groups have studied the effects of 1-butyl-3-methylimidazolium-based ILs ([BMIM^+^] ILs) with different anions on insulin properties. Takekiyo et al. found that [BMIM^+^] thiocyanate, along with ammonium-based ILs, strongly inhibited the formation of insulin fibrils, where the specific interactions between ILs and particular amino acid residues were the main cause of fibrillization suppression [28]. In other work, Takekiyo et al. [29] and Ishikawa et al. [30] showed that ILs possess fibril-dissolving ability in a manner dependent on the cation, the anion, and their concentration, with imidazolium-based ILs having greater dissolving ability than ammonium-based ILs. Ishikawa et al. reported >80% recovery of insulin monomer secondary structure [30]. Kumar and Venkatesu found that [BMIM^+^] chloride and [BMIM^+^] bromide stabilized insulin to some extent, but [BMIM^+^] ILs with thiocyanate, hydrogen sulfate, acetate, and iodide completely denatured insulin under the studied conditions [31]. Todinova et al. studied the thermal stability and secondary structure preservation of insulin. They found that [BMIM^+^] acetate, [BMIM^+^] trifluoroacetate, [BMIM^+^] dicyanamide and [BMIM^+^] chloride preserved helical structure and stabilized denaturation temperature to some degree compared to IL-free medium. In contrast, [BMIM^+^] thiocyanate and [BMIM^+^] tricyanomethanide had a destabilizing effect [32].

Despite a growing number of studies, the proven method for predicting the effect of particular ILs on protein stability or formulating the general mechanism of amyloid aggregation in ILs is still missing. The outcome of the protein–IL interaction is based on a particular combination of cation/anion/protein properties and specific cation–protein, anion–protein, and cation–anion interactions, where water or additional solvent must be considered. Experimental conditions also play a non-negligible role. For this reason, there is high demand for systematic studies to characterize the mechanism of action of ILs and to recognize and define the relationship between the physicochemical properties of ILs and their effect on protein stability and amyloid aggregation. There are currently only a few similar works studying insulin in ionic liquids. Thus, by choosing and investigating insulin, we provide valuable data, filling the gap in the current knowledge.

In this study, we selected five water-miscible imidazolium-based ILs consisting of 1-ethyl-3-methylimidazolium cation [EMIM^+^] in combination with hydrogen sulfate [HSO_4_^−^], acetate [AC^−^], chloride [Cl^−^], nitrate [NO_3_^−^], and tetrafluoroborate [BF_4_^−^] anions to assess the potential impact of Hofmeister effect on insulin amyloid aggregation. The Hofmeister series ranks ions according to their effect on various protein properties, solubility, stability, activity, and crystallization. Therefore, the potential involvement of this phenomenon was also considered to participate in amyloid fibrillization. However, while many studies have shown that the Hofmeister effect plays a role in promoting/inhibiting protein amyloid fibrillization, others have reported partial or no correlation with the Hofmeister series [33]. We studied the effect of the chosen aqueous IL solutions on the fibrillization kinetics, structure, and morphology of human insulin amyloid fibrils using ThT fluorescence assay, AFM, and FTIR spectroscopy. We found that, despite the different kosmotropicity/chaotropicity of anions, all of the studied ILs were able to promote insulin amyloid aggregation in a manner dependent on the concentration and the anion type of the ILs. The extent of the effects followed the reverse Hofmeister series at higher concentrations, suggesting specific ion interactions of ILs with the protein surface. ILs also induced the formation of fibrils with different morphologies, depending on the anion.

## 2. Results and Discussion

### 2.1. Effect of Ionic Liquids on the Insulin Amyloid Fibrillization Process

**Figure 1:**
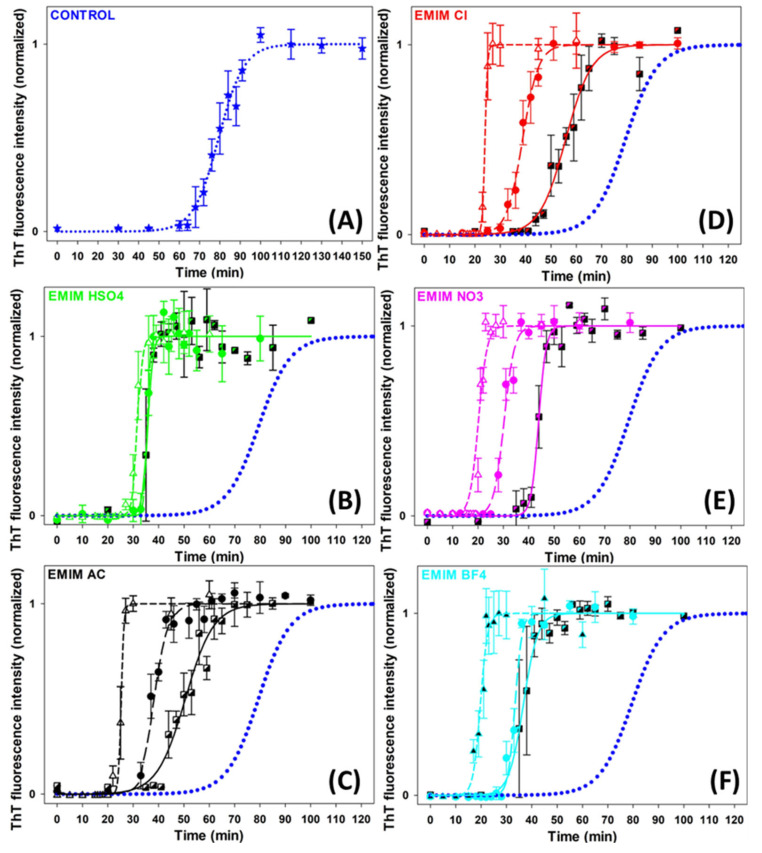
Graphic representation of insulin fibrillization in the absence (blue dotted line in (**A**–**F**)) and presence of (**B**) [EMIM^+^] [HSO_4_^−^], (**C**) [EMIM^+^] [AC^−^], (**D**) [EMIM^+^] [Cl^−^], (**E**) [EMIM^+^] [NO_3_^−^], (**F**) [EMIM^+^] [BF_4_^−^]. IL-concentrations: C_IL_ = 10 mM (semi-filled square, solid line); C_IL_ = 25 mM (circle, long-dash line); C_IL_ = 100 mM (open triangle, short-dash line).

The effects of five ILs consisting of [EMIM^+^] cation and five different anions of the Hofmeister series ([HSO_4_^−^], [AC^−^], [Cl^−^], [NO_3_^−^] and [BF_4_^−^]) (Figure S1) on insulin amyloid fibrillization were studied to assess the role of the Hofmeister effect in this process. The kinetics of the insulin amyloid fibrillization in the absence and presence of ILs at concentrations of 10 mM, 25 mM, and 100 mM was monitored by Thioflavin T (ThT) assay (Figure 1). The kinetic curves have a typical sigmoidal shape corresponding to the three-phased nucleation–elongation mechanism described by: (i) the lag phase (formation of the nuclei) characterized by lag time (t_lag_); (ii) the elongation phase (the polymerization of oligomers into fibrils) described by fibrillization half-time (t_half_) and aggregation rate constant (k_agg_); and (iii) the plateau phase (accumulation of mature fibrils). All obtained parameters are summarized in Table 1.

In the absence of ILs, the growth curve of insulin amyloid fibrils (blue dotted line, Figure 1) was characterized by kinetics parameters t_lag_~67 min, t_half_~79 min, and k_agg_~0.16 min^−1^. The addition of all studied ILs significantly accelerated the fibril formation at all used concentrations in a concentration-dependent manner. The lag time and fibrillization half-time were reduced by 30–55% at an IL concentration of 10 mM and by 55–75% at higher IL concentrations. The dose-dependent shortening of t_lag_ suggests that ILs favor nuclei formation. Moreover, the reduced t_half_ and increased k_agg_ values indicate accelerated fibril elongation in all cases. We also studied the effect of 250 mM ILs, but the obtained parameters were burdened by more than 20% error due to the very short lag times and the high steepness of the curves.

The anions used in this study are usually ordered with respect to their protein solubility effect as follows: [HSO_4_^−^] > [AC^−^] > [Cl^−^] > [NO_3_^−^] > [BF_4_^−^]. The anions on the left side of [Cl^−^] are kosmotropes, possessing the tendency to suppress unfolding. In contrast, those on the right side are chaotropes, anions that promote protein denaturation. The [Cl^−^] anion is considered a neutral borderline anion. When comparing the parameters of each IL, it can be concluded that at lower IL concentrations, the presence of [AC^−^] shortened the lag phase and fibrillization half-time to a greater extent than [Cl^−^], while the effect of [HSO_4_^−^] was stronger than the effect of [AC^−^], [Cl^−^] and [NO_3_^−^]. However, a correlation between the kinetics parameters and the position of the anions in the Hofmeister series was observed at higher concentrations. At an ILs concentration of 100 mM, the chaotropic anions, [BF_4_^−^] and [NO_3_^−^], caused a more pronounced shortening of the lag phase and fibrillization half-time compared to the neutral anion [Cl^−^] and the kosmotropic anions [AC^−^] and [HSO_4_^−^].

The efficiency of the anions used to shorten the t_lag_ and t_half_ follows the so-called electroselectivity series: [HSO_4_^−^] < [AC^−^] < [Cl^−^] < [NO_3_^−^] < [BF_4_^−^]. The electroselectivity series scales inversely with the Hofmeister series for monovalent ions [34]. The ranking of the efficiency of used anions suggests that the ions might specifically interact with polar and charged groups on the protein surface [35]. These results are in agreement with previous findings that the stability and amyloid fibrillization of proteins in IL environments are modulated by several factors, namely electrostatic screening along with preferential exclusion or direct binding of ions of ILs with the charged protein surface [36]. Since the insulin in pH~1.6 is positively charged, long-range electrostatic shielding is generally expected to be the main factor affecting the amyloid aggregation in the ILs environment. The interplay between long-range non-specific interactions, the Hofmeister effect (preferential interactions of ions with water), and specific protein–ion interactions are determining factors in promoting/inhibiting the amyloid aggregation of proteins [35,37,38].

One of the important factors facilitating globular protein amyloid aggregation is the formation of partially unfolded non-native conformations. Although the [AC^−^] and [HSO_4_^−^] anions are considered kosmotropic, thus suppressing the protein unfolding, they accelerated the insulin amyloid fibrillization in our study. The [AC^−^] anion is known to dissociate insufficiently from the [EMIM^+^] cation, producing populations of charged ionic and neutral groups even at low pH. The amphiphilic nature of the acetate may contribute to the non-specific interactions with the non-polar groups on the protein surface, interfering with hydrophobic intermolecular contacts responsible for aggregation. Kumar and Venkatesu [31] found that 1-butyl-3-methylimidazolium [BMIM^+^] ILs with strongly hydrated kosmotropic anions [AC^−^] and [HSO_4_^−^] acted as denaturants of insulin, while [BMIM^+^] [Cl^−^] was identified as a stabilizer of the insulin structure in some extent. However, Todinova et al. reported insulin destabilization at pH 2 and elevated temperature [32]. Contrary to our results, Takekiyo et al. [28] found that ILs containing [NO_3_^−^] anion combined with ethylammonium or propylammonium cations suppressed the insulin amyloid aggregation, and reported that the affinity between ILs and specific amino acid residues in insulin was found to be the main cause for the suppression of insulin amyloid formation.

These results show that the effect of selected ILs on insulin amyloid fibrillization is driven by specific protein–anion interactions only at a concentration of 100 mM. At lower concentrations, the ion–water interactions and non-specific long-range electrostatic shielding may be taken into account.

### 2.2. Effect of Ionic Liquids on the Morphology of Insulin Amyloid Fibrils

**Figure 2:**
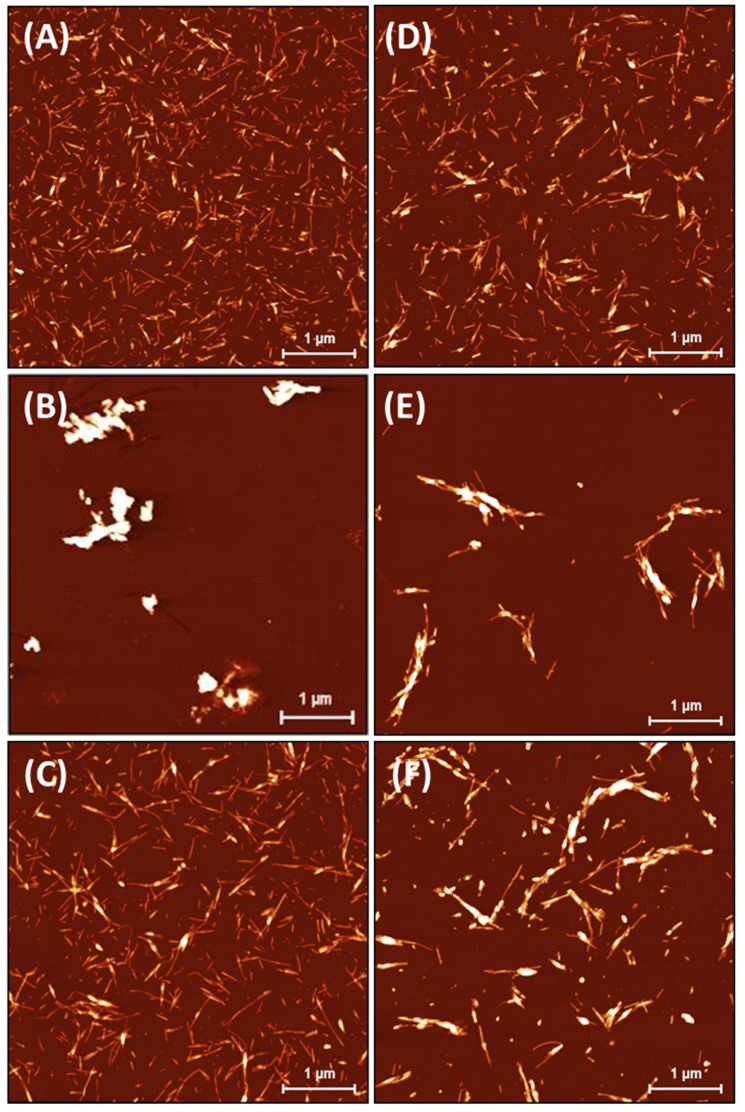
Insulin amyloid fibrils formed in absence of ILs (**A**) and with 25 mM IL solutions of (**B**) [EMIM^+^] [HSO_4_^−^], (**C**) [EMIM^+^] [AC^−^], (**D**) [EMIM^+^] [Cl^−^], (**E**) [EMIM^+^] [NO_3_^−^], and (**F**) [EMIM^+^] [BF_4_^−^].

**Figure 3:**
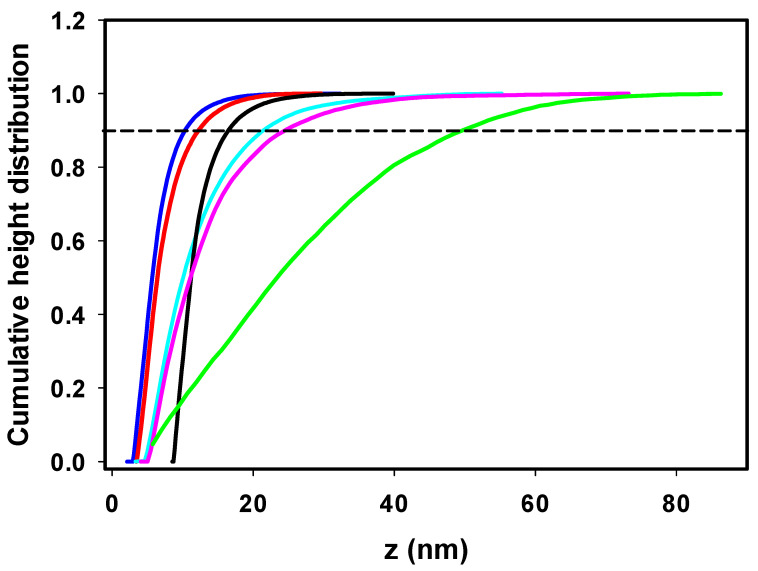
Cumulative height distribution of insulin fibrils prepared in the absence of ILs (blue) and with 25 mM IL solutions of [EMIM^+^] [HSO_4_^−^] (green); [EMIM^+^] [AC^−^] (black); [EMIM^+^] [Cl^−^] (red); [EMIM^+^] [NO_3_^−^] (magenta); [EMIM^+^] [BF_4_^−^] (cyan).

The insulin fibrils formed in the absence/presence of ILs were visualized using atomic force microscopy (AFM). The highest morphological variability of fibrils was observed at an ILs concentration of 25 mM, as shown in Figure 2. At an ILs concentration of 100 mM, the fibrils tended to associate into large clusters not suitable for further image analysis (Figure S2). The incubation of insulin under fibrillization-inducing conditions (see Section 3.2) in the absence of ILs resulted in the formation of a large number of shorter, fragmented amyloid fibrils with needle-like fibril morphologies (Figure 2A). This is a typical insulin fibrillar morphology that has been observed in several other studies. Nielsen et al. found that insulin fibrils formed at pH 1.6 in HCl or acetic acid consisted of twisted fibrils, 15–25 nm in diameter, clustered in parallel bundles when agitated during formation. Similar but less twisted laterally arranged thicker bundles were observed in acetic acid containing 0.1 M NaCl [39], suggesting that adding salts can significantly change the morphology of fibrils. In our case, the presence of kosmotropic [HSO_4_^−^] led to the formation of large amyloid fibril clusters (Figure 2B). A similar morphology to that of the IL-free insulin fibrils was observed in the presence of [EMIM^+^] [AC^−^] and [EMIM^+^] [Cl^−^] (Figure 2C,D). The insulin in the presence of ILs with chaotropic anions [NO_3_^−^] and [BF_4_^−^] formed longer fibrils with an increased tendency for lateral association (Figure 2E,F). The observed differences in fibril morphology urged us to perform the quantitative analysis using Gwyddion image analysis software. The calculated cumulative height distributions (Figure 3) were used to obtain the z_90%_ parameter, indicating the value below which 90% of the objects in the AFM image have their maximal height. The z_90%_ parameters were 10.36 ± 0.15 nm for the IL-free insulin fibrils, and 12.37 ± 0.16 nm and 16.56 ± 0.59 nm for insulin fibrils formed in the presence of [Cl^−^] and [AC^−^] ILs, respectively. The addition of chaotropic anion ILs increased the z_90%_ values of insulin fibril to 21.58 ± 0.77 nm for [BF_4_^−^] and 24.84 ± 0.42 nm for [NO_3_^−^], respectively. These results indicate that chaotropic anions induced the formation of fibrils with a higher height, mainly due to the lateral association and overlapping, while fibrils formed in [Cl^−^] and [AC^−^] ILs are more comparable with IL-free fibril’s morphology. The largest z_90%_ of 50.89 ± 1.02 nm was calculated for IL with kosmotropic [HSO_4_^−^]. This is an interesting result, considering the kosmotropicity of [HSO_4_^−^]. However, our data from kinetics measurements indicate that a 25 mM concentration of [HSO_4_^−^] causes the shortening of t_lag_, t_half_, and the elongation time necessary for the formation of long protofibrils from oligomers with the highest values of k_agg_ = 1.519. Based on these data, we assume that insulin molecules in the presence of 25 mM [HSO_4_^−^] IL form nuclei and, subsequently, oligomers very quickly, but the elongation of those structures into highly organized structures is even faster than in the case of other ILs and IL-free insulin, leading to the formation of large clusters.

### 2.3. Effect of Ionic Liquids on the Structure of Insulin and Insulin Amyloid Fibrils

**Figure 4:**
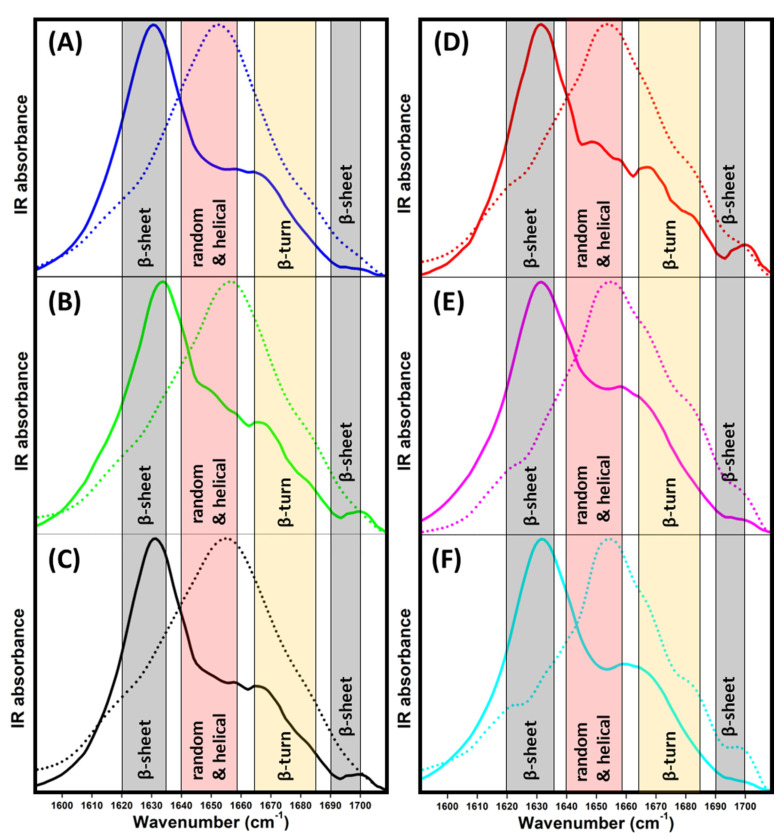
ATR-FTIR spectra of insulin (dotted line) and insulin fibrils (solid line) in the (**A**) absence (blue) and presence of 25 mM ILs solutions of (**B**) [EMIM^+^] [HSO_4_^−^] (green), (**C**) [EMIM^+^] [AC^−^] (black), (**D**) [EMIM^+^] [Cl^−^] (red), (**E**) [EMIM^+^] [NO_3_^−^] (pink), (**F**) [EMIM^+^] [BF_4_^−^] (cyan).

ATR-FTIR measurements were performed in the amide I region to examine the secondary structure of insulin and insulin fibrils to better understand the nature of the differences in the morphologies of the obtained fibrils observed using AFM. The spectrum of insulin in the absence of ILs (Figure 4A, blue dotted line) clearly shows that insulin occurs in a predominantly α-helical structure (~55%) with a portion of β-turn (~24%) and intramolecular β-sheet (~20%) structures (Table 2). In the presence of ILs, the overall shape of spectra remained partially preserved (Figure 4B–F dotted lines). The impact of the presence of ILs is more clearly visible in the second-derivative spectra (Figure S3A) and the content of particular protein secondary structures (Table 2) calculated from the deconvolved spectra (Figures S4 and S5).

The presence of ILs induces an increase in β-turn content at the expense of the α-helical and intramolecular β-sheet structures, with the most prominent changes being caused by chaotropic [NO_3_^−^] and [BF_4_^−^] ILs. The ILs containing kosmotropic anions [AC^−^] and [HSO_4_^−^] also induced similar structural changes with a weaker effect on insulin secondary structure content, manifesting the ability of all ILs to facilitate insulin amyloid aggregation. The decrease in the ordered structure to slightly loose β-turns enhanced the formation of the partially unfolded structure, which was more prone to aggregation, as indicated by the kinetic data. Moreover, at elevated temperature, the presence of ILs presumably enabled the exposure of amyloidogenic regions (partial unfolding) as a consequence of direct interactions with insulin molecules and ion–solvent interactions, thus perturbing the insulin hydration layer [40].

During insulin fibrillization, the α-helical structures are converted to β-sheets characteristic for the formation of mature amyloid fibrils (Figure 4, solid line). IL-free insulin forms fibrils with high β-sheet content (~58%) and only a ~14% α-helical structure content. Interestingly, the effect of ILs on the overall secondary structure content in insulin fibrils was negligible compared to that of IL-free insulin (Table 2). These data suggest that the secondary structure content in all formed insulin fibrils with 25 mM ILs is similar, and is not dependent on the presence of ILs and the type of anion. This is a relatively surprising result, as we observed the formation of distinct morphologies of insulin fibrils in the presence of 25 mM of different ILs, especially in the case of [HSO_4_^−^]-containing ILs. We suggest that all ILs are able to enhance the formation of nuclei, oligomers, and protofibrils, but with a similar internal structural organization, resulting in mature fibrils with similar secondary structure content, but different morphology.

The observed similarity in the FTIR spectra of amyloid fibrils indicates that the non-specific long-range electrostatic screening may be responsible for the formation of oligomers/protofibrils with similar secondary structure arrangements transferred to mature fibrils. The difference in morphology is partially modulated by the kosmotropicity/chaotropicity of the anions, and also correlates with the fibrillization rate and the gap between t_half_ and t_lag_ values. The kinetic curves for the formation of amyloid fibrils in the presence of the [NO_3_^−^] and [BF_4_^−^] anions show the shortest t_lag_ and t_half_, and a similar k_agg_ (within the standard deviation), although those anions induce the formation of longer, laterally associated fibrils. We can speculate that they are weakly hydrated low-charge-density anions that can non-specifically interact with apolar regions of protein. These interactions can enhance the unfolding and reduce the energy associated with hydrating the exposed hydrophobic regions facilitating the fibrillization, followed by the lateral association of fibrils. The presence of strongly hydrated high-charge-density anions [HSO_4_^−^] induces the fibrillization with the fastest aggregation rate and the smallest gap between t_lag_ and t_half_ values. The ability of strongly hydrated anions may modulate the hydration layer near the protein surface, leading to the lateral association of fibrils. However, another kosmotropic anion, [AC^−^], exerts a similar effect on morphology as the weakly chaotropic [Cl^−^], with similar kinetic parameters. The amphiphilic nature of the [AC^−^] anion, along with the weaker dissociation form of the [EMIM^+^] cation, can interfere with hydrophobic intermolecular contacts responsible for aggregation.

## 3. Materials and Methods

### 3.1. Chemicals

Human insulin (lyophilized powder, recombinant, expressed in yeast) and Thioflavin T (ThT) were purchased from Sigma Aldrich (St. Louis, MO, USA). The ionic liquids 1-ethyl-3-methylimidazolium hydrogensulphate ([EMIM^+^] [HSO_4_^−^]), 1-ethyl-3-methylimidazolium acetate ([EMIM^+^] [AC^−^]), 1-ethyl-3-methylimidazolium chloride ([EMIM^+^] [Cl^−^]), 1-ethyl-3-methylimidazolium nitrate ([EMIM^+^] [NO_3_^−^]) and 1-ethyl-3-methylimidazolium tetrafluoroborate ([EMIM^+^] [BF_4_^−^]) were purchased from IoLiTec (Heilbronn, Germany). All chemicals were of analytical reagent grade.

### 3.2. Insulin Amyloid Fibril Formation In Vitro

Insulin amyloid fibrils were prepared by dissolving insulin in an aqueous solution containing (0 mM, 10 mM, 25 mM, 100 mM and 250 mM) ILs to a final insulin concentration of 20 µM. The pH was adjusted to 1.6 using a small amount of 1 M HCl before each experiment and carefully checked at every experimental step. Ultrapure deionized water (Milli Q, Merck KGaA, Darmstadt, Germany) was used for the experiments. The prepared protein solutions were distributed into 200 μL test tubes (sample volume 100 μL) and incubated in a thermo-mixer under fibrillization-inducing conditions at 50 °C with constant stirring (1200 rpm). The formation of insulin fibrils was monitored using a ThT fluorescence assay and visualized by atomic force microscopy (AFM).

### 3.3. Thioflavin T (ThT) Fluorescence Assay

ThT is a fluorescent probe that specifically interacts with the cross-β-sheet-rich structures of amyloid fibrils accompanied by a significant increase in ThT fluorescence intensity upon excitation at λ_EX_ = 440 nm with an emission maximum at λ_EM_ = 485 nm. The insulin samples for the ThT assay were taken from a thermo-mixer at different time points. ThT was added to reach the final 40 μM concentration, followed by incubation at 37 °C for 60 min in the dark. All ThT fluorescence intensity measurements were performed in a Synergy Mx (BioTek, Winooski, VT, USA) spectrofluorimeter in a 96-well plate with an excitation wavelength set at 440 nm and emission was recorded at 485 nm. The emission and excitation slits were set at 9.0/9.0 nm, and the top probe vertical offset was 4 mm.

### 3.4. Kinetics of Insulin Fibrillization

A ThT fluorescence assay was used to establish the fibrillization kinetics of insulin (20 μM) in the presence of 0 mM, 10 mM, 25 mM, 100 mM and 250 mM ILs. The insulin samples were incubated at fibrillization-inducing conditions and withdrawn at given time points. All ThT fluorescence experiments were performed in triplicate. The resulting data represent the average of three individual measurements, and the error bars depict the average deviation. The time dependences of ThT fluorescence emission intensity were fitted with Boltzmann sigmoidal function using the following equation:

(1) $$y=y_{1}+\;\frac{y_{2}\;-\;y_{1}}{1\;+\;exp\left(-\;\frac{2\left(t\;-\;t_{half}\right)}{t_{half}\;-\;t_{lag}}\right)}$$

where *y* is the fluorescence intensity, *y***_1_** and *y***_2_** are the initial and final values of fluorescence intensity, *t* is the time, *t_half_* denotes the fibrillization half-time (time at 50% of fluorescence maximum), and *t_lag_* denotes lag-phase duration. The aggregation constant *k_agg_* was calculated as *k_agg_* = 2/(*t_half_* − *t_lag_*). The fluorescence intensity was normalized using the parameters of the fitted curves.

### 3.5. Atomic Force Microscopy (AFM)

The samples of (mature) insulin fibrils were diluted for AFM purposes at a ratio of 1:8 (fibrils to acidic water) and applied dropwise (20 μL) on the surface of freshly cleaved mica followed by 5 min adsorption. Then, the samples (mica) were rinsed with ultra-pure water and left to dry (on air, at room temperature). AFM images of 1024 × 1024 pix resolution and 5 × 5 μM in size were taken using a Scanning Probe Microscope (Veeco di Innova, Bruker, Billerica, MA, USA) in a tapping mode under ambient conditions with SNL-10 (silicone tip on nitride lever coated with 4 nm Ti/Au layer) cantilever. Unfiltered AFM images were processed and analyzed using Gwyddion 2.60 software (Department of Nanometrology, Czech Metrology Institute, Brno, Czech Republic) [41].

### 3.6. Attenuated Total Reflectance–Fourier-Transform Infrared (ATR-FTIR) Spectroscopy

ATR-FTIR spectra of native insulin and insulin fibrils were measured using a Nicolet™ 8700 Fourier-transform infrared spectrometer (Thermo Fisher Scientific, Waltham, MA, USA) equipped with Smart OMNI-Sampler. Insulin with a concentration of 5 mg/mL was dissolved in ILs with a concentration of 25 mM. The samples were then left to equilibrate for 10 min in the test tube. Insulin fibrils prepared under fibrillization-inducing conditions (insulin concentration 20 μM) were concentrated by centrifugation (for a detailed centrifugation protocol, see the Supplementary Materials). A 5 uL sample droplet was applied (dropwise) onto the sampler surface and left to dry for 20 min at room temperature before scanning. The spectra for each sample were obtained by collecting and averaging 128 scans with a resolution of 2 cm^−1^ over a range of 1600 to 1700 cm^−1^. All spectra were baseline-corrected. Data processing was performed in OriginPro software 2023 (OriginLab Corporation, Northampton, MA, USA). The recorded spectra were smoothed by applying a Savitzky–Golay filter (with a 20-point window) and normalized. Deconvolution of the smoothed normalized spectra was performed using the Peak Analyzer function. Peak positions were set in compliance with the raw spectra. Data were fitted using the Gaussian peak function followed by integration of resulting curves, providing % content of present secondary structures. The assignment of peaks to the corresponding secondary structures was performed with respect to published literature.

## 4. Conclusions

In this work, we explored the effects of five ILs consisting of 1-ethyl-3-methylimidazolium cation [EMIM^+^] in combination with hydrogen sulfate [HSO_4_^−^], acetate [AC^−^], chloride [Cl^−^], nitrate [NO_3_^−^], and tetrafluoroborate [BF_4_^−^] anions on the kinetics of insulin amyloid fibrillization and the morphology of the obtained fibrils. We found that, regardless, of the kosmotropicity/chaotropicity of anions, all ILs promoted the amyloid fibrillization of insulin at all of the studied concentrations (10 mM, 25 mM, 100 mM), as demonstrated by a decrease in the lag time and fibrillization half-time parameters. At a concentration of 100 mM, the efficiency of anions in promoting insulin amyloid aggregation followed the electroselectivity series [HSO_4_^−^] < [AC^−^] < [Cl^−^] < [NO_3_^−^] < [BF_4_^−^], indicating the involvement of specific interactions of ions with the protein surface. We observed the formation of large clusters of fibrils under these conditions, disabling further image analysis.

The dependence of the kinetics parameters of insulin fibrillization on the electroselectivity series observed at an IL concentration of 100 mM disappeared at an IL concentration of 25 mM.

The kosmotropic [HSO_4_^−^] and [AC^−^] showed a stronger effect than the chaotropic [NO_3_^−^] or neutral [Cl^−^], respectively. The lack of correlation can be attributed to the changed balance in competition between screening effect, preferential exclusions (ion–water, protein–water interactions), and specific protein–ion interactions. The presence of 25 mM [HSO_4_^−^] ILs led to the formation of large amyloid fibril clusters, while the presence of [AC^−^] and [Cl^−^] led to the formation of fibrils with a similar needle-like morphology to that of the IL-free insulin fibrils. The insulin in the presence of the ILs with chaotropic anions [NO_3_^−^] and [BF_4_^−^] formed longer fibrils with an increased tendency for lateral association. Even though we observed a different fibril morphology to be induced by 25 mM ILs, the secondary structure content was relatively similar for insulin in ILs under the initial conditions before fibrillization. Our results indicate that at an IL concentration of 100 mM, the promotion of insulin amyloid fibrillization is predominantly driven by specific interactions with the protein surface. At an IL concentration of 25 mM, the effect of specific interactions is weakened at the expense of electrostatic screening and ion impact on protein–water and ion–water interactions. For weakly hydrated [NO_3_^−^], [Cl^−^], or [BF_4_^−^] anions, the effect may be shifted toward electrostatic screening, while for highly hydrated [HSO_4_^−^], the bulk ion–solvent and protein–ion–solvent interactions play a role.

In conclusion, our work revealed that the studied ILs were able to accelerate insulin amyloid fibrillization and produced fibrils with different morphologies. These results may contribute to a better understanding of the non/involvement of the Hofmeister effect in IL-induced amyloid fibrillization. Our findings may be beneficial as the first step towards further utilization of amyloid fibrils in biotechnological applications.

## Figures and Tables

**Table 1:** The kinetics parameters of insulin fibrillization derived from the kinetics shown in Figure 1.

Medium	t_lag_ (min)	t_half_ (min)	k_agg_ (min^−1^)
Control	66.93 ± 1.65	79.42 ± 0.92	0.160 ± 0.024
EMIM HSO_4_ [10 mM]	33.61 ± 0.69	35.71 ± 0.38	0.952 ± 0.301
EMIM HSO_4_ [25 mM]	34.17 ± 1.28	35.48 ± 0.43	1.519 ± 1.024
EMIM HSO_4_ [100 mM]	29.14 ± 0.15	31.09 ± 0.06	1.028 ± 0.069
EMIM AC [10 mM]	39.39 ± 2.13	50.53 ± 1.20	0.179 ± 0.030
EMIM AC [25 mM]	32.00 ± 1.14	37.66 ± 0.53	0.353 ± 0.060
EMIM AC [100 mM]	24.18 ± 0.21	25.28 ± 0.09	1.822 ± 0.419
EMIM Cl [10 mM]	45.46 ± 1.85	55.78 ± 0.99	0.194 ± 0.039
EMIM Cl [25 mM]	32.49 ± 0.73	38.79 ± 0.36	0.318 ± 0.041
EMIM Cl [100 mM]	22.91 ± 0.05	23.94 ± 0.04	1.951 ± 0.119
EMIM NO_3_ [10 mM]	40.95 ± 0.79	43.92 ± 0.33	0.672 ± 0.158
EMIM NO_3_ [25 mM]	26.18 ± 0.72	30.29 ± 0.42	0.487 ± 0.086
EMIM NO_3_ [100 mM]	16.74 ± 2.21	20.16 ± 1.09	0.585 ± 0.238
EMIM BF_4_ [10 mM]	31.01 ± 1.12	36.69 ± 0.42	0.352 ± 0.057
EMIM BF_4_ [25 mM]	30.62 ± 0.82	33.40 ± 0.30	0.718 ± 0.174
EMIM BF_4_ [100 mM]	16.85 ± 0.98	19.73 ± 0.46	0.695 ± 0.170

**Table 2:** Calculated content of secondary structures of insulin and insulin fibrils.

	INSULIN			INSULIN FIBRILS		
Peak Center Position	1640–1658 cm^−1^	1620–1635 & 1690–1700 cm^−1^	1665–1685 cm^−1^	1640–1658 cm^−1^	1620–1635 & 1690–1700 cm^−1^	1665–1685 cm^−1^
Secondary Structure	Random & α-Helix (%)	β-Sheet (%)	β-Turn (%)	Random & α-Helix (%)	β-Sheet (%)	β-Turn (%)
Control	53.7	19.6	24.4	14.4	58.6	21.2
[EMIM^+^] [HSO_4_^−^]	47.3	15.1	34.5	14.0	55.1	21.8
[EMIM^+^] [AC^−^]	44.8	18.4	35.0	12.9	59.8	23.1
[EMIM^+^] [Cl^−^]	46.0	19.0	30.7	13.1	59.6	23.3
[EMIM^+^] [NO_3_^−^]	46.2	13.7	35.8	20.0	51.7	24.1
[EMIM^+^] [BF_4_^−^]	40.7	15.9	38.2	13.1	57.0	26.3

## Data Availability

Data will be available on reasonable request.

## Supplementary Materials

The following supporting information can be downloaded at: https://www.mdpi.com/article/10.3390/ijms24119699/s1.
