# Supplementary material for: Modulation of Insulin Amyloid Fibrillization in Imidazolium-Based Ionic Liquids with Hofmeister Series Anions
Source: Int J Mol Sci. 2023 Jun 2;24(11):9699. doi: 10.3390/ijms24119699 (PMC10253814; doi:10.3390/ijms24119699)
Supplement: Supplementary file 1 [file ijms-24-09699-s001.zip › ijms-2435059-supplementary.pdf]

International Journal of *Molecular Sciences*

Supplementary material

SUPPLEMENT

CENTRIFUGATION PROTOCOL

The centrifugation process was performed using *Amicon® 10K* filters. The procedure is as follows: (I) concentrating the sample – (II) removing the filtrate – (III) resuspending the concentrated sample with control solution [ultrapure water pH 1.6] (i.e., buffer exchange) – (IV) re-concentrating the sample. This procedure may be repeated until the acceptable buffer threshold is reached. The manufacturer assumes a 5% sample loss at 100-fold concentration. Filter adsorption depends on the sample concentration, pH, temperature, and duration of sample-filter contact.

The removal of IL solutions from fibril samples (containing 20 μM insulin fibrils) and concentrating fibrils to achieve higher value for ATR-FTIR measurements was performed in the following steps:

1) 500 μL of the sample solution was placed into the filter, followed by centrifugation (spinning) for 15 minutes at 14 000 rcf (concentrated sample stays in the filter)
2) 500 μL of the sample solution was added to the concentrate, followed by centrifugation for 15 minutes at 14 000 rcf (this step was repeated 3-times)
3) 300 μL of control solution was added to the sample concentrate (resuspension), followed by centrifugation for 15 minutes at 14 000 rcf
4) the obtained final concentrate (~50 μL) in the filter was invertedly centrifuged for 1 minute at 1000 rcf (i.e., transferring the sample into the test tube)

During the process, the IL solutions were removed from fibril samples (buffer exchange).

IONIC LIQUID	SCHEMATIC REPRESENTATION<br>CATION ANION
[EMIM$^+$] [HSO$_4^-$]<br><br>IUPAC: 1-ethyl-3-methylimidazol-3-ium hydrogen sulfate	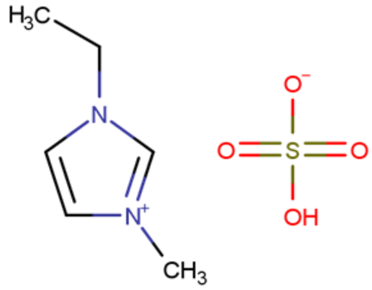
[EMIM$^+$] [AC$^-$]<br><br>IUPAC: 1-ethyl-3-methylimidazol-3-ium acetate	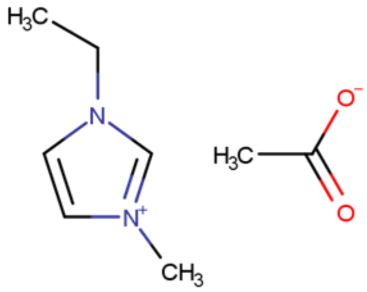
[EMIM$^+$] [Cl$^-$]<br><br>IUPAC: 1-ethyl-3-methylimidazol-3-ium chloride	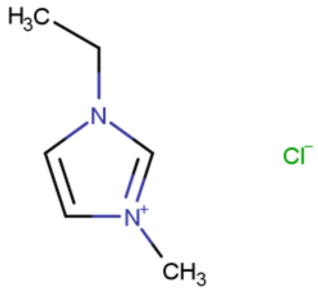
[EMIM$^+$] [NO$_3^-$]<br><br>IUPAC: 1-ethyl-3-methylimidazol-3-ium nitrate	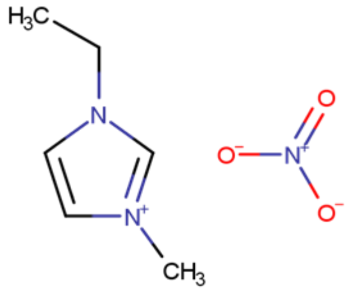
[EMIM$^+$] [BF$_4^-$]<br><br>IUPAC: 1-ethyl-3-methylimidazol-3-ium tetrafluoroborate	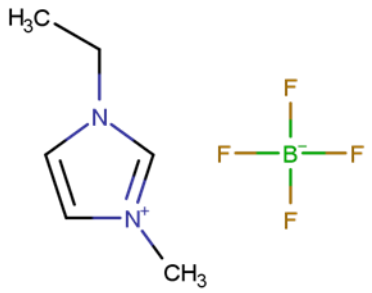

Figure S1. Schematic representation of studied ionic liquids.

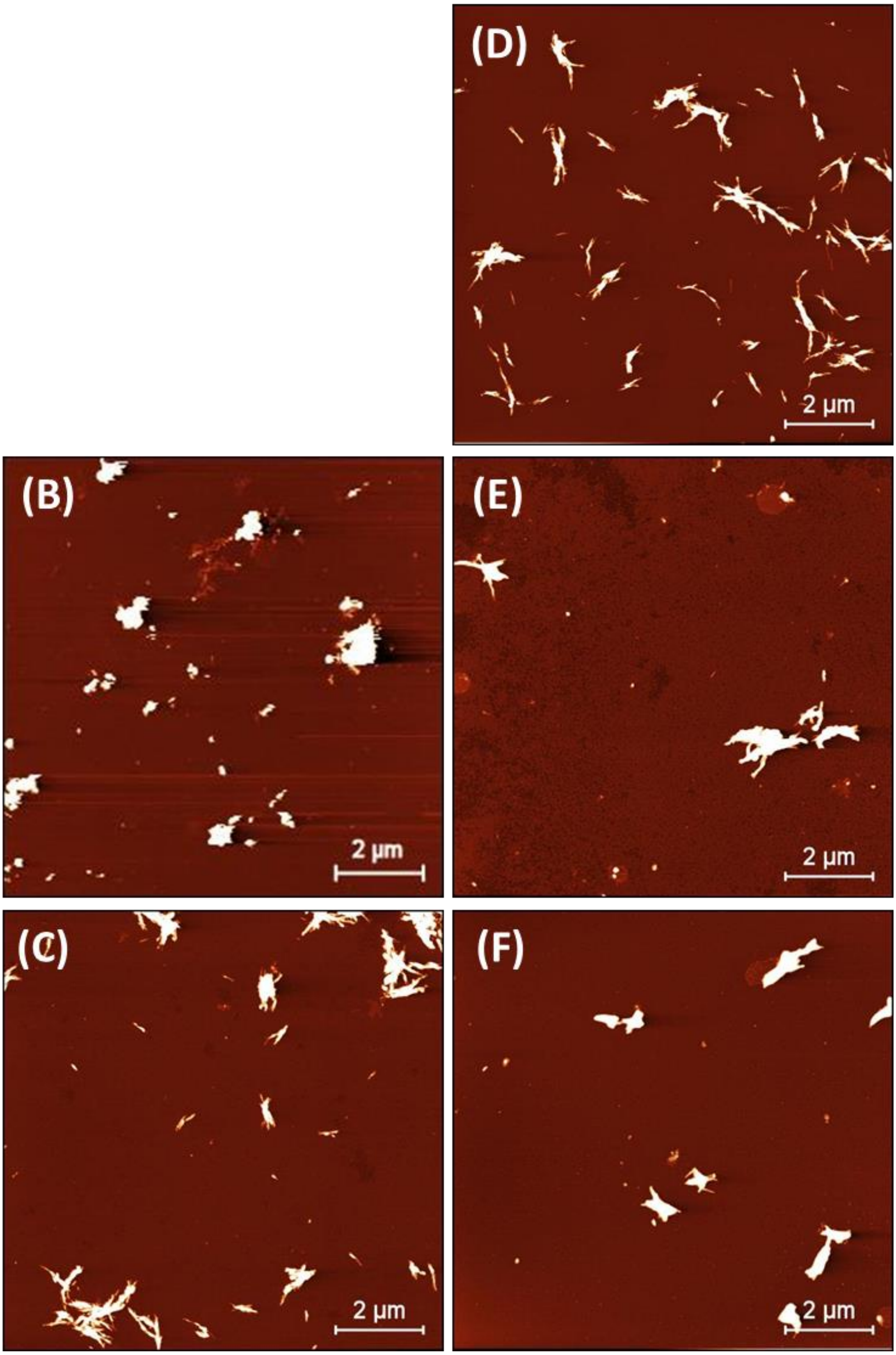

Figure S2. AFM images of insulin fibrils formed in the presence of 100 mM ILs: (B) EMIM HSO4 100 mM, (C) EMIM AC 100 mM, (D) EMIM Cl 100 mM, (E) EMIM NO3 100 mM, (F) EMIM BF4 100 mM.

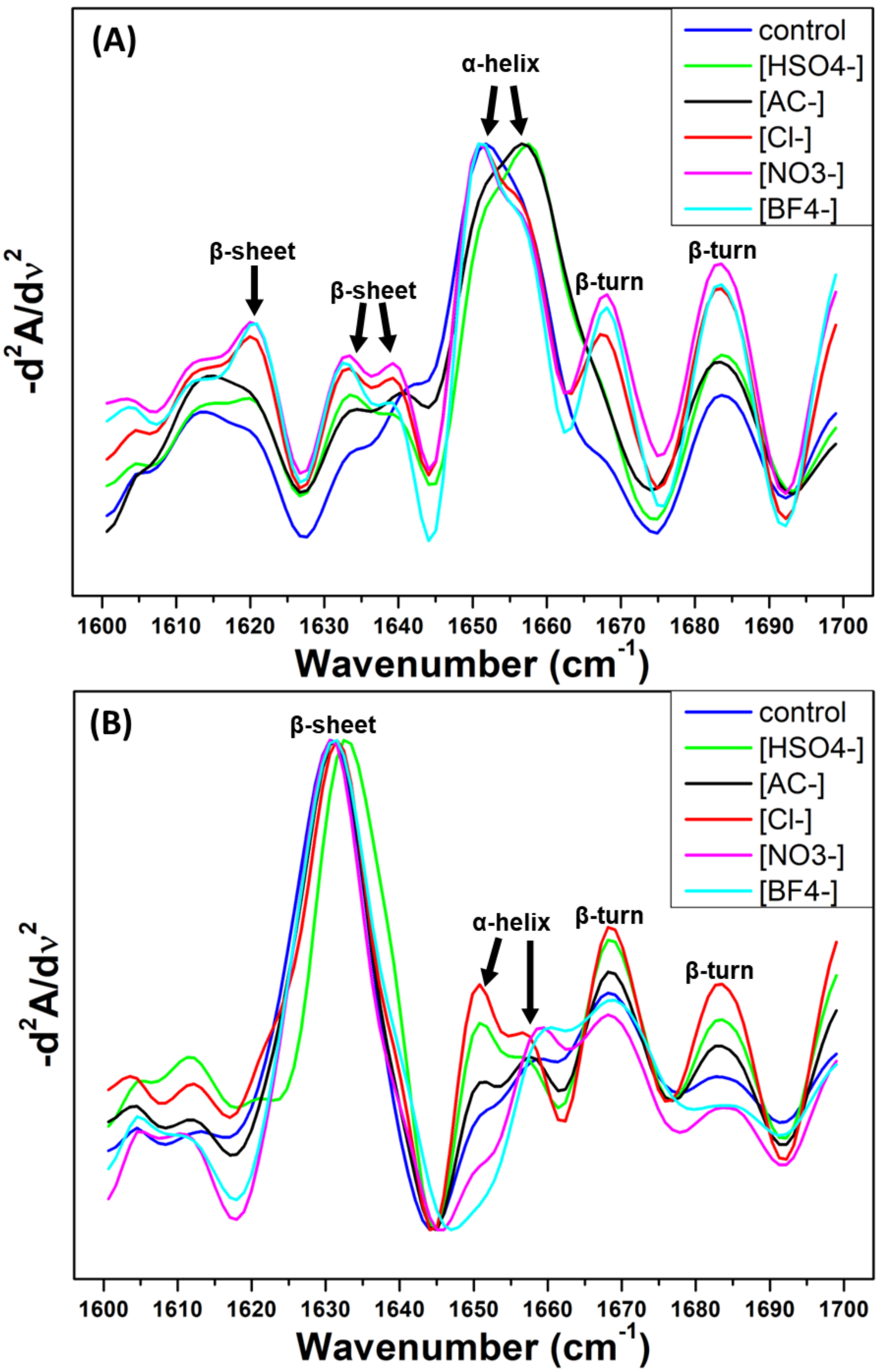

Figure S3. Second derivative of the Amide I band of (A) insulin and (B) insulin amyloid fibrils. Frequencies were assigned to particular corresponding structures according Yang[1].

---

[1] 1. Yang, H.; Yang, S.; Kong, J.; Dong, A.; Yu, S. Obtaining Information about Protein Secondary Structures in Aqueous Solution Using Fourier Transform IR Spectroscopy. *Nat. Protoc.* **2015**, *10*, 382–396, doi:10.1038/nprot.2015.024.

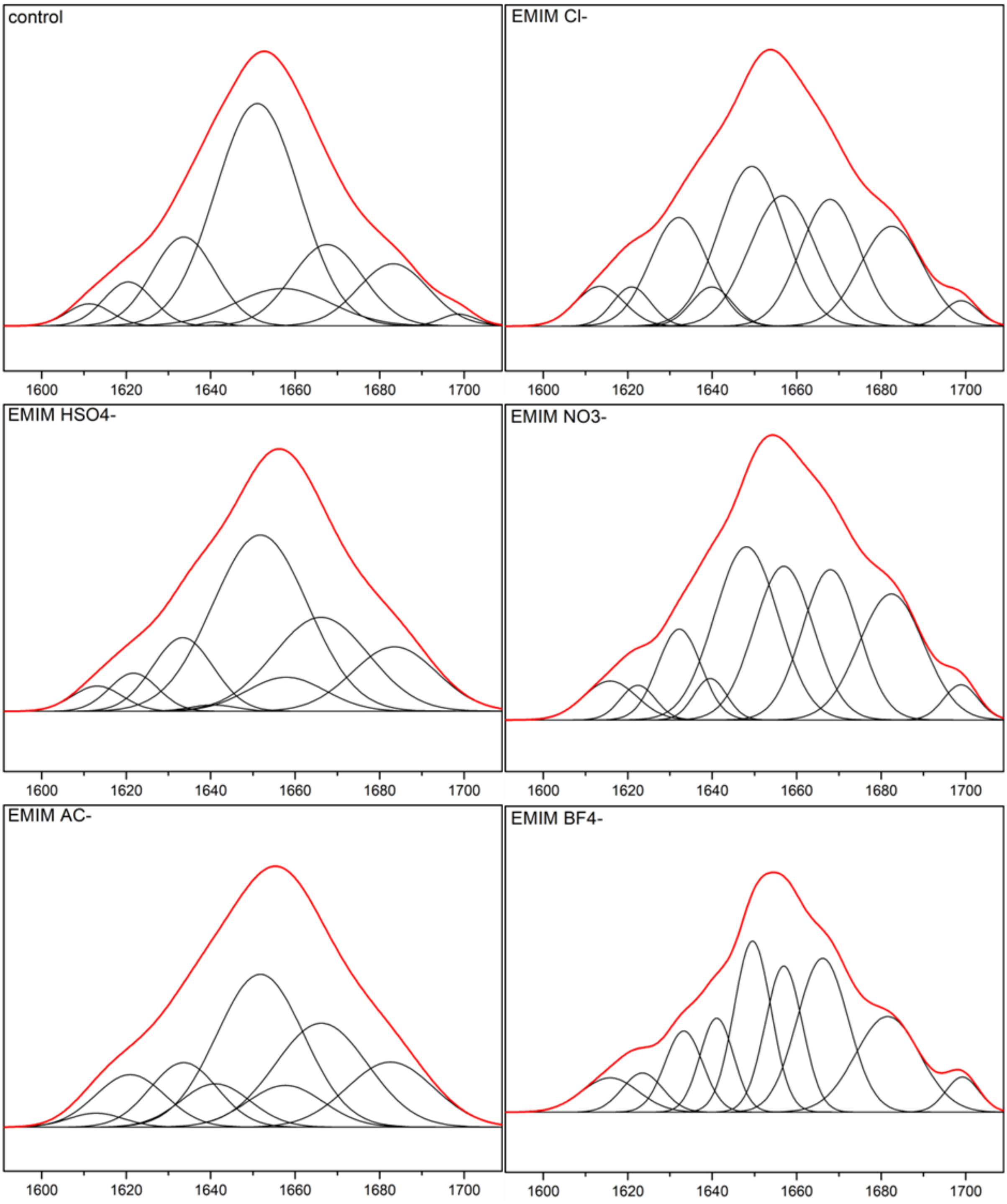

Figure S4. Deconvolved spectra of insulin with individual peaks shown.

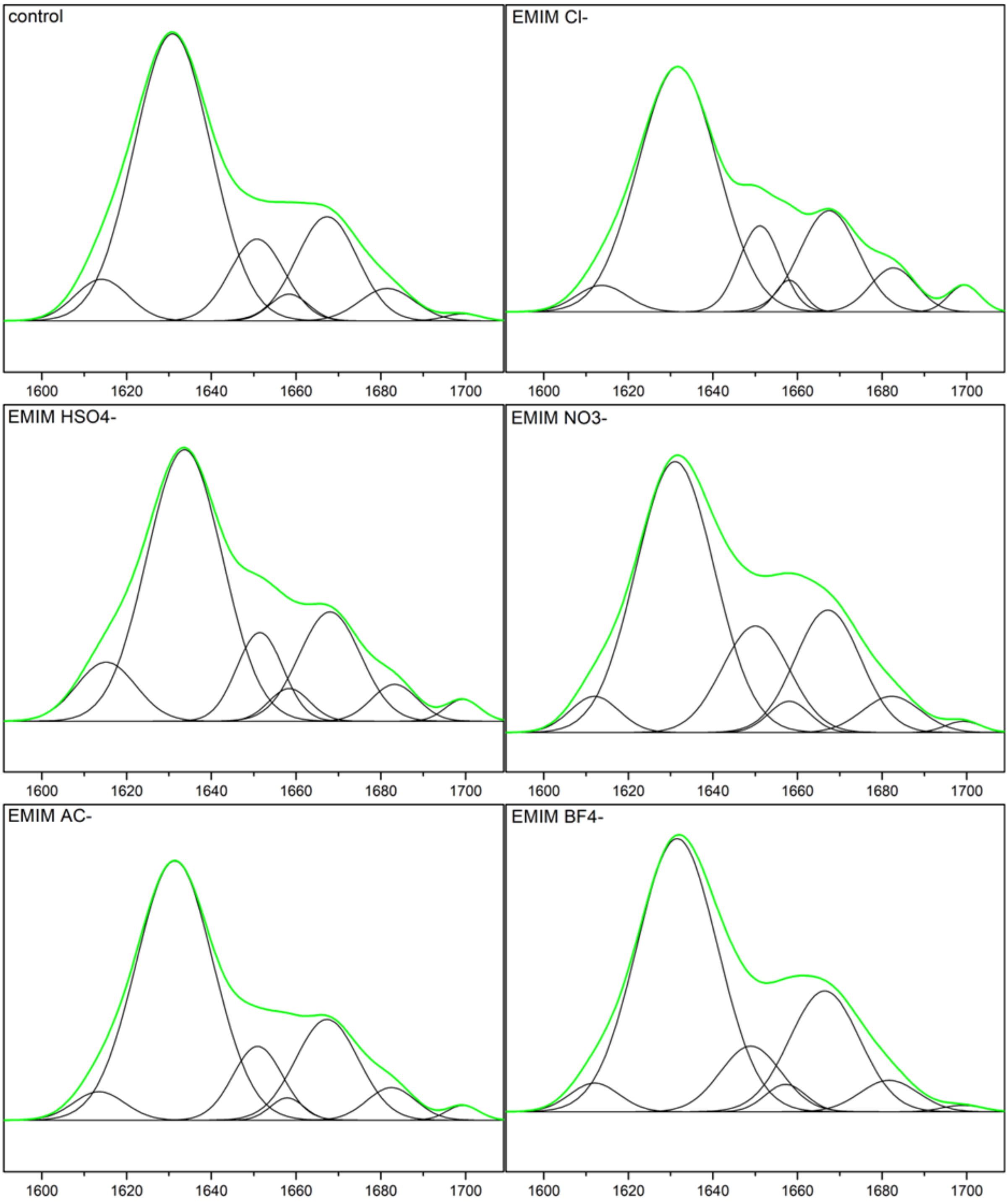

Figure S5. Deconvolved spectra of insulin fibrils with individual peaks shown.
